# A New Horned Crocodile from the Plio-Pleistocene Hominid Sites at Olduvai Gorge, Tanzania

**DOI:** 10.1371/journal.pone.0009333

**Published:** 2010-02-24

**Authors:** Christopher A. Brochu, Jackson Njau, Robert J. Blumenschine, Llewellyn D. Densmore

**Affiliations:** 1 Department of Geoscience, University of Iowa, Iowa City, Iowa, United States of America; 2 Human Evolution Research Center, Department of Integrative Biology, University of California, Berkeley, California, United States of America; 3 National Natural History Museum, Arusha, Tanzania; 4 Center for Human Evolutionary Studies, Department of Anthropology, Rutgers University, New Brunswick, New Jersey, United States of America; 5 Department of Biological Sciences, Texas Tech University, Lubbock, Texas, United States of America; Institute of Evolutionary Biology (CSIC-UPF), Spain

## Abstract

**Background:**

The fossil record reveals surprising crocodile diversity in the Neogene of Africa, but relationships with their living relatives and the biogeographic origins of the modern African crocodylian fauna are poorly understood. A Plio-Pleistocene crocodile from Olduvai Gorge, Tanzania, represents a new extinct species and shows that high crocodylian diversity in Africa persisted after the Miocene. It had prominent triangular “horns” over the ears and a relatively deep snout, these resemble those of the recently extinct Malagasy crocodile *Voay robustus*, but the new species lacks features found among osteolaemines and shares derived similarities with living species of *Crocodylus*.

**Methodology/Principal Findings:**

The holotype consists of a partial skull and skeleton and was collected on the surface between two tuffs dated to approximately 1.84 million years (Ma), in the same interval near the type localities for the hominids *Homo habilis* and *Australopithecus boisei*. It was compared with previously-collected material from Olduvai Gorge referable to the same species. Phylogenetic analysis places the new form within or adjacent to crown *Crocodylus*.

**Conclusions/Significance:**

The new crocodile species was the largest predator encountered by our ancestors at Olduvai Gorge, as indicated by hominid specimens preserving crocodile bite marks from these sites. The new species also reinforces the emerging view of high crocodylian diversity throughout the Neogene, and it represents one of the few extinct species referable to crown genus *Crocodylus*.

## Introduction

Until recently, it was thought that the ancestors of modern African crocodiles would be found among Oligocene through Pliocene fossils found in Africa [1], [2], [3], [4]. Many of these resembled the living Nile crocodile (*Crocodylus niloticus*), but recent phylogenetic analyses argue instead that many belong to an endemic clade with only one unambiguous living representative – the African dwarf crocodile *Osteolaemus*. Gross similarity with *C. niloticus*, along with misconceptions of crocodiles as evolutionarily static “living fossils,” obscured the diversity of this group through the Neogene of Africa, Madagascar, and possibly Aldabra Atoll and the Arabian Peninsula [5], [6], [7], [8], [9], [10], [11], [12], [13], [14], [15], [16], [17]. Just as living African crocodile species may represent cryptic species complexes [18], [19], [20], their fossil relatives were more diverse than previously supposed, with outwardly similar (though not always related) species mistaken for geographically widespread species with long stratigraphic ranges.

Several questions remain. Fossil and molecular data suggest a Neogene divergence among living species of *Crocodylus*, and they usually support a close relationship between the *C. niloticus* and a clade of Neotropical species [21], [22], [23], [24], [25], but relationships among other species of *Crocodylus* are largely unresolved, as is the placement of the African sharp-nosed crocodile *(Mecistops cataphractus*), which may be related to either *Crocodylus* or *Osteolaemus* [23], [25], [26], [27], [28], [29]. Thus, whether *C. niloticus* represents an African lineage separate from the osteolaemine radiation or a more recent immigrant is unclear [30]. A better understanding of Neogene African crocodylids is needed to resolve these issues.

One of these, *Rimasuchus lloydi*, was long thought to be close to the ancestry of *C. niloticus* before phylogenetic analyses suggested an osteolaemine affinity [17], [23]. But codings in these analyses are based on material from the Middle Miocene type locality in Egypt, and fossils from all over Africa, ranging in age from the Early Miocene through Quaternary, have been referred to *R. lloydi* [2], [13], [15], [16], [31], [32]. The phylogenetic relationships of these other fossils remain untested.

Some of these are from the Plio-Pleistocene deposits exposed in Beds I through IV at Olduvai Gorge, northern Tanzania. Bed I is the oldest level at Olduvai and is best known for key discoveries of extinct human species, including the holotypes of *Australopithecus boisei* and *Homo habilis*, as well as evidence of the earliest stone tools [33], [34], [35]. Some of these hominids were bitten by crocodiles at or near the time of death [36], [37], and some objects thought to be early tools may be crocodile gastroliths [38]. The crocodiles were referred first to *C. niloticus* [39] and later to *Rimasuchus lloydi* [2].

A partial skull and skeleton collected in 2007 by the Olduvai Landscape Paleoanthropology Project prompted a reevaluation of crocodile remains from Olduvai Gorge. It reveals a deep-snouted, horned animal outwardly similar to a recently-extinct osteolaemine from Madagascar (*Voay robustus*) but referable to *Crocodylus*. It can be distinguished from other known species of *Crocodylus*, living or extinct, and forms the basis for a new species.

### Institutional Abbreviations

AMNH, American Museum of Natural History, New York; FMNH, Field Museum, Chicago; KNM, National Museums of Kenya, Nairobi; NHM, Natural History Museum, London; NNHM-OLD, National Natural History Museum, Arusha, Tanzania (Olduvai Collections); PNCZ, Parque Nacional Ciénaga de Zapata, Playa Larga, Matanzas, Cuba; USNM, U.S. National Museum of Natural History, Washington, DC.

### Anatomical Abbreviations

4t, 4th trochanter of femur; an, angular; art, articular; asf, anterior sacral facet; bo, basioccipital; ccr, caviconchal recess; cor, coronoid; cqc, cranioquadrate canal; cr, recesses on caviconchal recess medial wall; d, dentary; dlc, deltoid crest; dp, diapophysis; dpc, deltopectoral crest; ect, ectopterygoid; emf, external mandibular fenestra; en, external naris; eoa, external otic aperture; ex, exoccipital; f, frontal; faa, articular foramen aereum; faq, quadrate foramen aereum; fioc, foramen intermedius oralis caudalis; fm, foramen magnum; gf, glenoid fossa of articular; gfs, scapular glenoid fossa; hyp, hypapophysis; ibc, constriction on psterior iliac blade; if, incisive foramen; itf, infratemporal fenestra; j, jugal; k, keel; l, lacrimal; lc, lacrimal crest; lcf, lateral carotid foramen; leu, lateral Eustachian foramen; lf, lingual foramen; lhc, lateral hemicondyle; lp, lateral lamina of articular on surangular; m.pfp, medial process, prefrontal pillar; m5, fifth maxillary tooth/alveolus; mg, Meckelian groove; mhc, quadrate medial hemicondyle; mjf, medial jugal foramen; msc, muscle attachment scar; mx, maxilla; n, nasal; o, orbit; oc, occipital condyle; op, odontoid process; p.m5, protuberance on dorsal surface of maxilla corresponding to 5th alveolus; pal, palatine; pf, prefrontal; pfp, prefrontal pillar; pmx, premaxilla; pnr, prenarial rostrum; po, postorbital; pob, postorbital bar; poz, postzygapophysis; prz, prezygapophysis; psf, preotic siphonial foramen; psf, posterior sacral facet; pt, pterygoid; q, quadrate; qj, quadratojugal; sa, surangular; soc, supraoccipital; sof, suborbital fenestra; sp, splenial; sq, squamosal; stf, supratemporal fenestra; sym, symphysis; ta, posteriormost (terminal) alveolus; tp, transition point between dorsal surface of skull table and squamosal horn; vf, vagus foramen; xii, foramen for hypoglossal nerve (cranial nerve 12). Articulation surfaces for adjacent bone denoted with “s.” (e.g. articulation surface for the maxilla = s.mx).

### Nomenclatural Acts

The electronic version of this document does not represent a published work according to the International Code of Zoological Nomenclature (ICZN), and hence the nomenclatural acts contained in the electronic version are not available under that Code from the electronic edition. Therefore, a separate edition of this document was produced by a method that assures numerous identical and durable copies, and those copies were simultaneously obtainable (from the publication date noted on the first page of this article) for the purpose of providing a public and permanent scientific record, in accordance with Article 8.1 of the Code. The separate print-only edition is available on request from PloS by ending a request to PloS ONE, 185 Berry Street, Suite 3100, San Francisco, CA 94107, USA along with a check for $10 (to cover printing and postage) payable to “Public Library of Science.”

In addition, this published work and the nomenclatural acts it contains have been registered in ZooBank, the proposed online registration system for the ICZN. The ZooBank LSIDs (Life Science Identifiers) can be resolved and the associated information viewed through any standard web browser by appending the LSID to the prefix “http://zoobank.org/”. The LSID for this publication is urn:lsid:zoobank.org:pub:CB77D4ED-B0B6-4F16-AAE7-231CF9F4DEBE.

Clade names follow currently-used phylogenetic definitions [40]. Although the definition of Crocodylidae is context-dependent based on the position of *Gavialis*, the new species would be a crocodylid regardless of context.

### Systematic Paleontology

Eusuchia Huxley 1873

Crocodylia Gmelin 1789, sensu Benton and Clark 1988

Crocodylidae Cuvier 1807

*Crocodylus anthropophagus*, new species

urn:lsid:zoobank.org:act:052051B8-6503-42B3-8D7A-9E7E49578401

#### Holotype specimen

**Figure 1:**
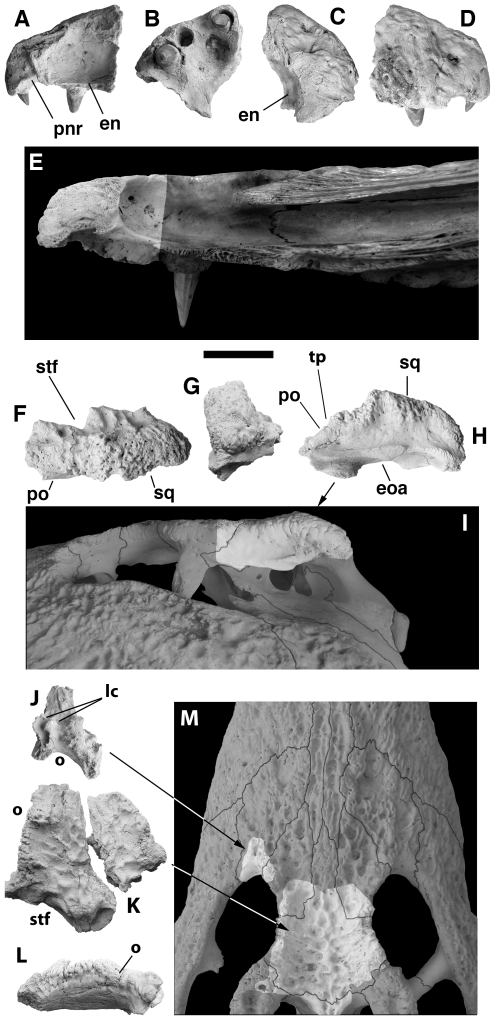
Cranial remains of NNHM-OLD-1001, holotype, *Crocodylus anthropophagus*, preserving features diagnostic of the species. Right premaxilla in medial (A), ventral (B), dorsal (C), and lateral (D) view; partial left squamosal in dorsal (F), posterior (G), and lateral (H) view; left lacrimal in dorsal view (J); frontal with adjoining parts of prefrontals in dorsal (K) and left lateral (L) view. Specimens are compared with Crocodylus niloticus (KNM OR44, E; AMNH 7136, right side reversed, I; KNM OR54, M). Scale = 1 cm.

**Figure 2:**
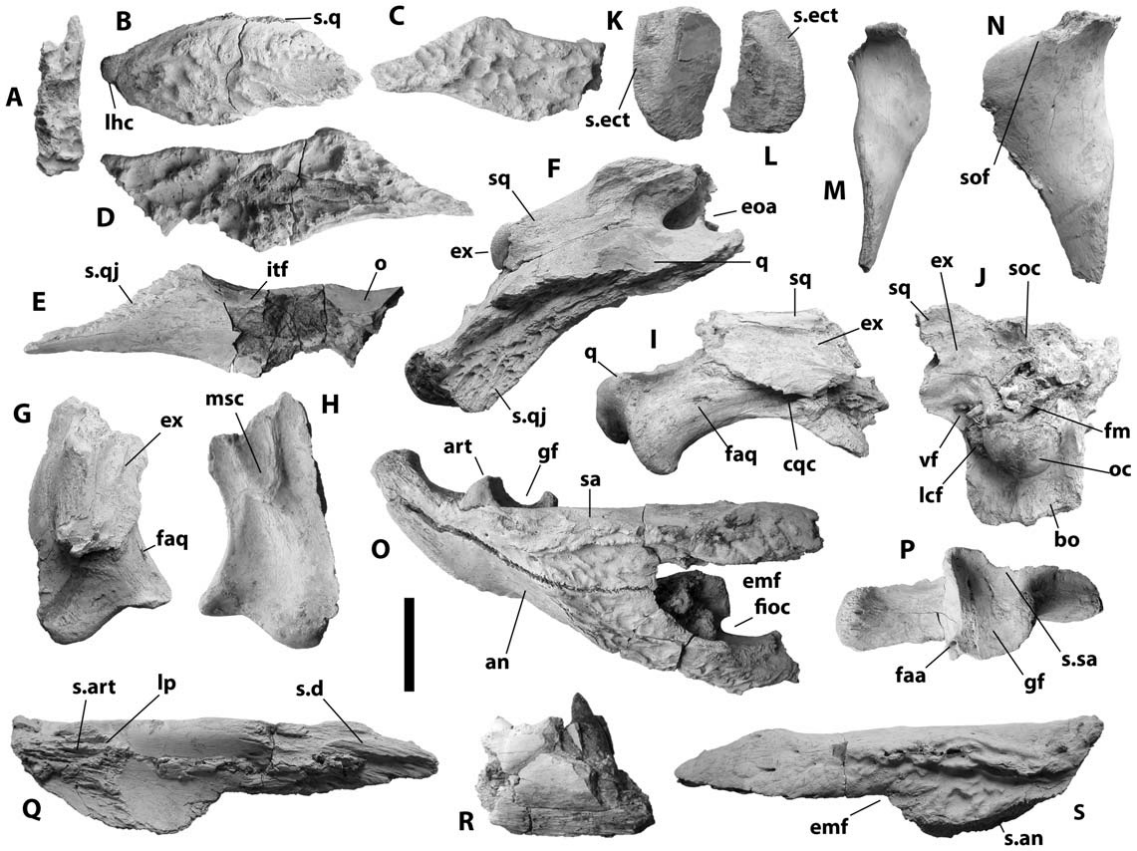
Craniomandibular remains of NNHM-OLD-1001, holotype, *Crocodylus anthropophagus*. A, partial left nasal, dorsal view; B, right quadratojugal, lateral view; C, right jugal, lateral view; D, left jugal, lateral view; E, left jugal, medial view; F, right otic region and quadrate ramus, lateral view; G, left quadrate ramus, dorsal view; H, left quadrate ramus, ventral view; I, left quadrate ramus and paroccipital process, posteromedial view; J, braincase, posterior view; K, right pterygoid wing, ventral view; L, left pterygoid wing, ventral view; M, right ectopterygoid, ventral view; N, left ectopterygoid, ventral view; O, right postdentary bones, lateral view; P, left quadrate, dorsal view; Q, left surangular, medial view; R, fragment of dentary; S, left surangular, lateral view. Scale = 5 cm.

**Figure 3:**
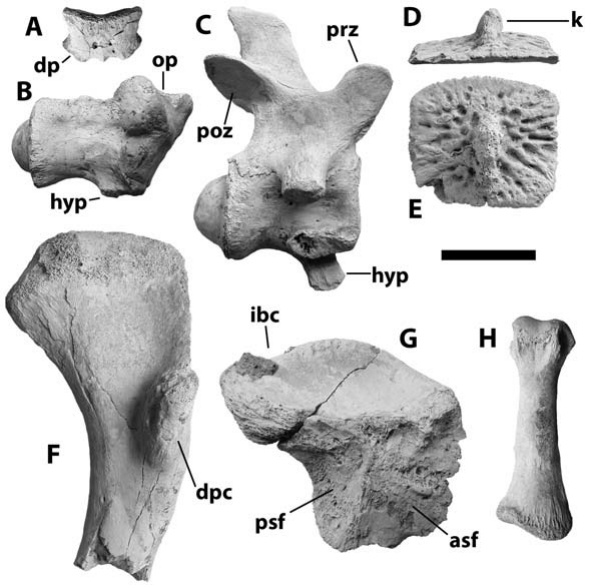
Postcranial remains of NNHM-OLD-1001, holotype, *Crocodylus anthropophagus*. A, atlas intercentrum, anterior view. B, axis centrum and odontoid process, right lateral view. C, cervical vertebra, right lateral view. D, dorsal osteoderm, posterior view. E, dorsal osteoderm, dorsal view. F, proximal half of left humerus, ventral view. G, left ilium, medial view. H, metapodial, dorsal view. Scale = 5 cm.

NNHM-OLD-1001, partial skull and skeleton (Fig. 1, Fig. 2, Fig. 3).

#### Referred Material

**Figure 4:**
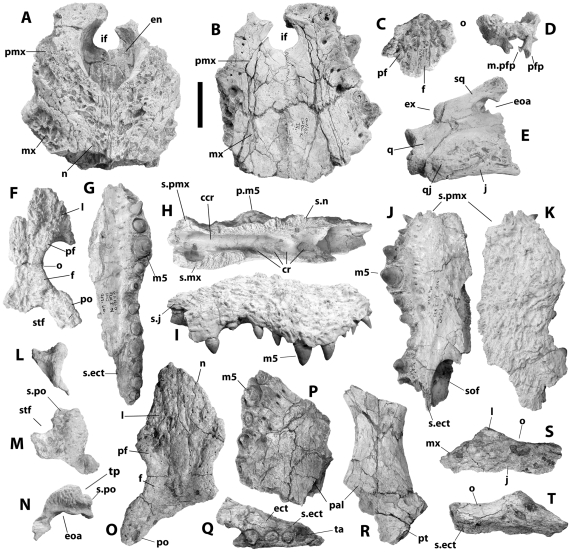
Cranial remains referred to *Crocodylus anthropophagus*. KNM CROC K OLD 62: anterior end of rostrum, dorsal (A) and ventral (B) view; partial frontal with portions of prefrontals in dorsal (C) and anterior (D) view; right otic region and quadrate ramus, lateral view (E). KNM FLKNI: partial orbital region, dorsal view (F); left maxilla, ventral view (G); right maxilla, medial (H), lateral (I), ventral (J), and dorsal (K) view; right squamosal, posterior (L), dorsal (M), and lateral (N) view. NHM R.5893: orbital region, dorsal view (O); partial right maxilla, ventral view (P); partial right maxilla and ectopterygoid, ventral view (Q); partial palatines and pterygoids, ventral view ( R); partial right jugal, lateral (S) and medial (T) view. Scale = 5 cm.

**Figure 5:**
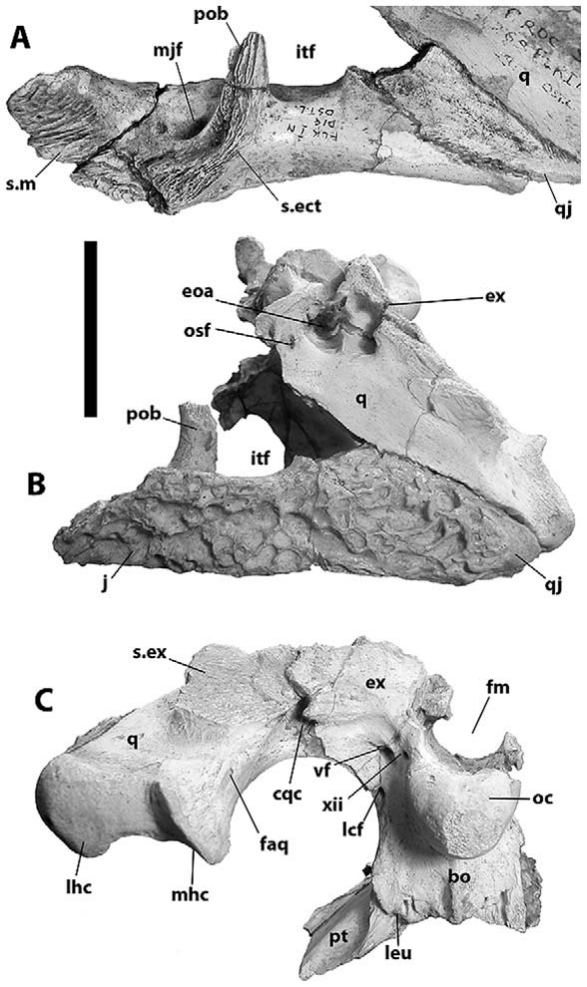
Partial braincase and left quadrate ramus of KNM FLKNI, *Crocodylus anthropophagus*, in medial (A), dorsolateral (B), and posterior (C) view. Scale = 5 cm.

NHM R.5891, cranial and postcranial fragments; NHM R.5893, partial skull and skeleton (Fig. 4O–T; Fig. 5D,E); NHM R.5894, postcranial elements; and several specimens in the KNM collections. Most do not have catalogue numbers beyond their collection date and locality. Postcranial elements cannot be associated with particular cranial material (or with each other), but all available cranial evidence suggests a single crocodylian species in these units. The following refer to particular specimens figured in this paper:

Crocodile Korongo (CROC K): OLD 62, partial skull (Fig. 4A–D); OLD 62 069/5866, right squamosal and quadrate ramus (Fig. 4E).

Bell's Korongo (BKII) channel: OLD 1960, right postdentary elements of mandible (Fig. 5A–B).

**Figure 6:**
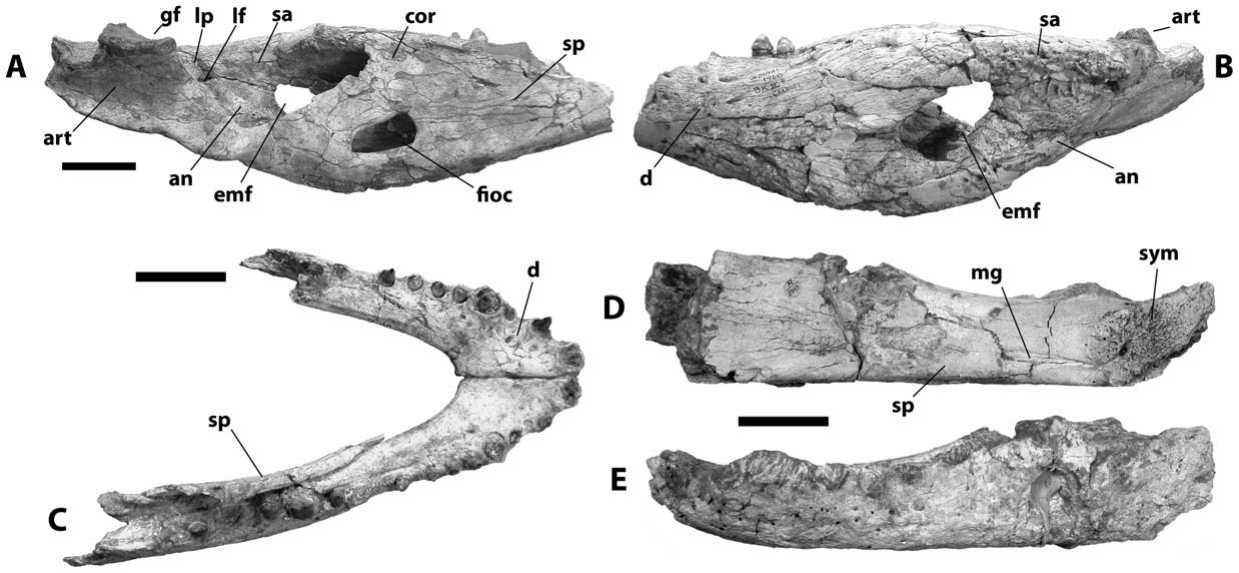
Mandibular remains referred to *Crocodylus anthropophagus*. KNM BKII OLD 1960: left postdentary bones and posterior end of dentary, medial (A) and lateral (B) view; KNM FLKNI, dentaries and portion of right splenial, dorsal view (C); NHM R.5893, left dentary and splenial, medial (D) and lateral (E) view. Scale = 5 cm.

**Figure 7:**
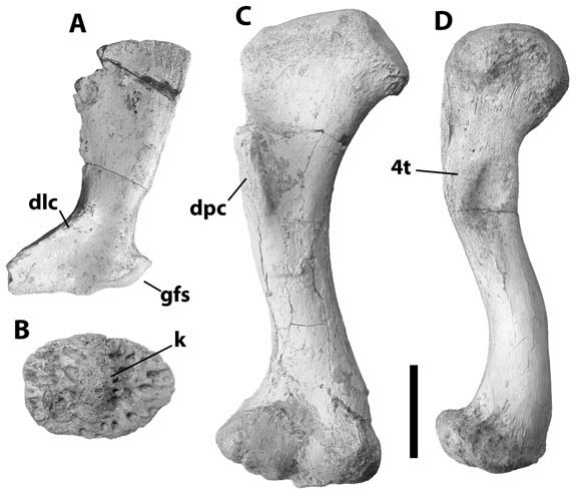
Postcranial material referred to *Crocodylus anthropophagus*. A, KNM DK I B, left scapula, lateral view; B, NHM R.5894, ?nuchal osteoderm; C, KNM DK I B OLD 62 54, right humerus, ventral view; D, KNM FLKNI, right femur, ventral view. Scale = 5 cm.

Frida Leakey Korongo North I (FLKNI): cranial, mandibular, and postcranial material (Fig. 4F–N, Fig. 5C, Fig. 6, Fig. 7D). These are derived from at least two (and probably more) individuals; the braincase (Fig. 6) is from a substantially smaller animal than most other cranial fragments.

Douglas Korongo, trench 1B (DK IB): scapula and humerus (OLD 62 54).

#### Etymology

*anthropos*, Greek, *human* and *phagos*, Greek, *eater*, in reference to the evidence that this animal included hominids in its diet.

#### Locality and Age

Plio-Pleistocene, Olduvai Gorge, northern Tanzania. The holotype was collected from the surface of Middle Bed I between Tuffs IB and IC, dated to 1.845+/−0.002 and 1.839+/−0.005 Ma, respectively [41]. FLKNI is near the type localities of *Australopithecus boisei* and *Homo habilis* and is from Upper Bed I. The DK locality also lies within Bed I. NHM R.5891 is from Bed I, and NHM R.5893 is from Bed II. Younger material from BK II (upper Bed II) and CROC K (Bed III or IV) is also referred to this species. Labels on KNM specimens from CROC K specify Bed IV, but published reports merely put crocodile remains from CROC K somewhere in Beds III or IV [35]. An additional specimen from Bed IV (NHM R.5892) may also pertain, but diagnostic features were not preserved. All of these predate the Holocene.

#### Diagnosis

*Crocodylus* with a prominent triangular projection (“horn”) at the posterolateral corner of each squamosal dorsal to otic aperture at maturity; projection has discrete boundaries in lateral and posterior view. Pair of thin crests on rostrum corresponding to the maxillary-nasal sutures. Maxillary ramus of ectopterygoid may not be forked, though expression of the cleft varies intraspecifically in most modern *Crocodylus*. External naris opens anterodorsally rather than dorsally. Lacks the elongate preorbital crest typical of Indo-Pacific *Crocodylus*, and lacks the median rostral boss diagnostic for Neotropical *Crocodylus*.

#### Description

The premaxillae (Figs. 1A–D and 4A,B) form the anterior and lateral margins of the narial aperture and are separated by the nasals medially behind the naris. Each bears an acute posterior process between the nasal and maxilla extending back to approximately the second maxillary alveolus. The naris opens anterodorsally, and the dorsal surface posterolateral to the narial rim and along the premaxillary-maxillary suture is inflated. The premaxillae surround a circular incisive foramen ventrally, and there is a deep occlusal pit anterolateral to the incisive foramen. The palatal lamina of each premaxilla has a convex posterior margin, causing the premaxilla-maxilla suture on the palate to form a shallow W.

The right premaxilla of the holotype preserves three complete alveoli and the anterior margin of a fourth (Fig. 1B). There is a diastema between the first and second, and the second is smaller than both the first and third. The fourth is incomplete, but was larger than the third. The second alveolus is sometimes crowded away by the third during ontogeny in *Crocodylus* [42], [43], but we do not believe this happened here; in crocodiles lacking the second alveolus, diastemata separate the three anteriormost alveoli, and the second remaining alveolus (originally the third) is similar in size to the first. Alveoli are imperfectly preserved on the KNM CROC K OLD 62 snout, but a small alveolus adjacent to the premaxilla-maxilla suture shows that *C. anthropophagus* had five premaxillary alveoli.

None of the preserved maxillae are complete. One partial left element (Fig. 4G) preserves a complete series of 13 alveoli, of which the fifth behind the premaxilla is the largest. The maxillary palate is vaulted anteriorly, and the first six alveoli extend ventral to the palatal ramus. A small pit at the back of the toothrow might be the remnant of a fourteenth alveolus that no longer held teeth. Occlusal pits for the dentary teeth lie between the first ten alveoli. KNM FLKNI indicates that the suborbital fenestra extended anteriorly to the level of the ninth maxillary alveolus (Fig. 4J), and assuming the ectopterygoid was adjacent to four maxillary alveoli (see below), the maxillary ramus lateral to the fenestra bore five alveoli.

An isolated right maxilla (KNM FLKNI, Fig. 4H–K) preserves the medial wall of the caviconchal recess, revealing a linear array of shallow pits. The circular posterior opening to the recess lateral to the nasopharyngeal duct is approximately medial to the eighth maxillary alveolus. The dorsal surface of the maxilla bears a prominent circular protuberance posterodorsal to the fifth alveolus. The surface expands dorsally parallel to the sutural contact with the nasal, forming a sharp linear crest.

Each nasal bears a short conical process extending into the narial aperture. The nasals flare posteriorly as they approach the posterior tips of the premaxillae, but the point at which their lateral margins adopt a parasagittal orientation is not preserved. They taper posteriorly where they pass adjacent to the lacrimals and prefrontals, forming short triangular processes separating the frontal from each prefrontal.

None is complete, but the preserved jugal fragments (Figs. 1B–E, 4S,T, 6A,B) collectively indicate the shape of the element. The anterior ramus is flat and passes laterally over the maxilla. It forms the ventral margin of the orbit and bears one or two large foramina between the medial surface and postorbital bar. The posterior ramus is dorsoventrally shorter and mediolaterally thicker, tapering to a point posteriorly. It forms the ventral margin and posteroventral corner of the infratemporal fenestra. The jugal component of the postorbital bar is hemicylindrical, bearing a crescentic articulation facet for the ectopterygoid and postorbital medially.

The lacrimal forms the anterior margin of the orbit. The outline is not completely preserved, but it extended further anteriorly than the prefrontal. An oval aperture on its posterior surface, within the orbital margin, indicates the posterior opening of the lacrimal duct. It connected with the jugal laterally.

The partial left lacrimal associated with the holotype (Fig. 1J) preserves a series of thin anteroposteriorly-oriented crests on its dorsal surface – a mediolaterally robust crest extending from the lacrimal-prefrontal suture at the orbital margin and two thinner crests lateral to a shallow groove extending from the orbit. The medial crest and dorsal groove are generally present in most crocodyliforms (including most *Crocodylus*), but the lateral crests are not. They are not apparent on the other specimens preserving portions of the lacrimal (e.g. KNM FLKNI, Fig. 4F; NHM R5893, Fig. 4O), but this could be preservational – none of these preserves much of the lacrimal lateral to the dorsal groove. Nevertheless, pending better information on variation, these features are only provisionally considered diagnostic for the species.

The prefrontal forms the anteromedial margin of the orbit and extends anteriorly to form an acute process between the nasal and lacrimal. Based on NHM R5893 (Fig. 4O), the anterior process extended approximately as far forward as the frontal. Its lateral margin, where it contacts the lacrimal, is concave. The descending processes forming the dorsal part of the prefrontal pillars are mediolaterally compressed structures, and the left descending process of KNM CROC K OLD 62 (Fig. 4D) bears a medial process that is constricted at its base and anteroposteriorly elongate medially.

The dorsal surface of the frontal between the orbits is flat (Figs. 1K, 4C,F,O). Its anterior process is sharply demarcated from the main frontal body, and the broad anterior process itself terminates at an acute point approximately at the same level as the anterior margins of the prefrontal and the orbit. The frontoparietal suture is imperfectly preserved, but the posterior surface of the frontal is convex, and the suture did not pass within the supratemporal fenestra.

Those portions of the prefrontal and frontal bordering the orbit are sharply upturned (Fig. 4D). On each side, they form a continuous robust lamina extending from the prefrontal-lacrimal contact to the frontal-postorbital suture. The medial crest on the lacrimal can be seen as a rostral continuation of this structure. The frontal-prefrontal suture changes orientation from mediolateral to anteroposterior at a right angle immediately medial to the lamina. Two prominent knobs extend dorsally from each lamina, one entirely on the prefrontal and another at the frontal-prefrontal contact. This is most apparent on the holotype (Fig. 1K).

The postorbital includes a broadly crescentic dorsal corpus and columnar descending process comprising the dorsal and, ventrally, the medial portion of the postorbital bar. In at least one specimen (e.g. NNHM-OLD-1001, Fig. 1H), it expands dorsally as it approaches the squamosal posteriorly; but another isolated squamosal (Fig. 4N) expands abruptly behind its sutural surface for the postorbital, suggesting that the dorsal surface of the postorbital in that specimen would have been more planar.

The squamosal forms the posterolateral margin of the supratemporal fenestra. The lateral and posterior margins of the fenestra are almost linear, intersecting at a nearly right angle (Figs. 1F, 4M). The squamosal and postorbital together form the roof of the external otic recess, and the cloverleaf-shaped otic aperture itself is bordered posterodorsally by the squamosal. The lateral squamosal groove for the ear flap musculature is dorsoventrally broad (Figs. 1F, 4N). The squamosal bears a flat ventrolateral ramus that forms the anterior surface of the paroccipital process.

The dorsolateral margin of the squamosal forms a prominent dorsal hornlike projection. This takes the form of a mediolaterally flattened lamina and is triangular in lateral view, with an apex dorsal to the otic aperture and posterolateral to the supratemporal fenestra. It arises abruptly from the dorsal surface of the skull table. The apex is sharp in the holotype, and the lateral squamosal groove is continuous with a sulcus on the lateral surface of the horn (Fig. 1F–H). Other specimens suggest a more rounded apex and a broadly convex lateral surface (Fig. 4L–N).

The parietal is incompletely known. Its articulation surface for the frontal is concave, and it did not contribute to the supratemporal fenestra. Whether its dorsal surface was flat is unknown, but it did not expand laterally as it approached the squamosal and, hence, did not contribute to the squamosal horn.

The quadratojugal lies between the jugal and quadrate. The ascending ramus is not completely preserved, but based on sutural surfaces on the quadrate and jugal, it formed nearly all of the posterior margin of the infratemporal fenestra, extending from just dorsal to the posteroventral corner to nearly to its dorsal apex; but whether it contacted the squamosal is unknown.

The anterior process of the palatine was broad and formed a U-shaped structure at its anteriormost extent at approximately the level of the seventh maxillary alveolus (Fig. 4P). Posteriorly, the conjoined palatines (Fig. 4R) constitute the floor of the nasopharyngeal duct and the medial margins of the suborbital fenestrae. There are no discrete processes or expansions of the palatine into the fenestral space.

Based on NHM R5893 (Fig. 4Q), the maxillary ramus of the ectopterygoid lies adjacent to four maxillary alveoli, possibly forming the medialmost wall of the posteriormost two alveoli. The anterior tip of the ramus appears to not be forked, although there is a modest concavity in its outline; the attachment scar for the ectopterygoid on the right maxilla of KNM FLKNI suggests the absence of an anterior cleft. The pterygoidal ramus (Fig. 2M,N) would have been fixed to the ventral surface of the pterygoid along the ventrolateral sides of the pterygoid wings.

The pterygoids met the palatines along a linear sutural contact anterior to (and not intersecting) the internal choana (Fig. 4R). The pterygoid wings were broad and dorsoventrally thin, with flat articulation surfaces for the ectopterygoids ventrolaterally (Fig. 2K,L). The choana is partially preserved on a KNM specimen from FLKNI, and although the pterygoid surface was slightly elevated around the aperture, there was no choanal “neck.” Posteriorly, each pterygoid bears a small triangular process adjacent to the basioccipital, anterior to the lateral Eustachian foramen (Fig. 5C).

Anteriorly, the quadrate forms the margin of the otic aperture and is pierced by a small circular preotic foramen (Figs. 2F, 4E, 5B). Its dorsolateral surface is smooth ventral to these openings, in marked contrast to the heavily pitted quadratojugal and jugal. The quadrate ramus bears a small foramen aereum on its dorsomedial surface, and the medial hemicondyle is dorsoventrally expanded relative to its lateral counterpart (Figs. 2G, 5C). There is a large muscle attachment tubercle on the ventral surface of the ramus (Fig. 2H).

Details of the lateral braincase wall, including morphology of the laterosphenoid and prootic, are not preserved. Based on sutural contacts on the ventral surface of the frontal, the laterosphenoid capitate processes were oriented anterolaterally.

The supraoccipital is likewise poorly known. Based on the holotype (Fig. 2J), it is triangular in posterior view, bearing sagittal crest that thickens dorsally. It would have been exposed on the skull table, but the shape of the dorsal exposure is not preserved.

The exoccipital formed the posterior portion of the paroccipital process, narrowing laterally from the post-temporal fenestra (Fig. 2I,J). The cranioquadrate canal opens along the ventral margin of the exoccipital, passing anteromedially between the exoccipital and quadrate. Medially, the exoccipitals meet at the midline dorsal to the foramen magnum and extend posteriorly dorsal to the occipital condyle, where each is pierced by one or two small foramina for the hypoglossal nerve. The descending process of each exoccipital lateral to the main basioccipital body was pierced by a large common foramen for the ninth through eleventh cranial nerves and the jugular vein (lateral to the foramen magnum) and a carotid foramen lateral to the occipital condyle.

The basisphenoid is unknown, but based on sutural surfaces on the basioccipital of NNHM-OLD-1001and KNM FLKNI, it would have formed an anteroposteriorly thin sheet ventral to the basioccipital. This sheet would have had a dorsoventrally short exposure on the posterior occipital surface based on the minimal distance between the ventral margins of the basioccipital and pterygoid (Fig. 5C).

The basioccipital bears a robust spherical occipital condyle projecting from a main body (Figs. 2J, 5C). The main body bears a sagittal crest, and the exoccipital descending processes did not contribute to the modest basioccipital tubera. Notches for the lateral Eustachian openings are nearly lateral to the circular median Eustachian foramen. The main body is wedge-shaped in lateral view.

No complete dentaries are preserved, but based on preserved specimens (Fig. 6), there were at least fourteen alveoli on each ramus. The fourth alveolus was enlarged, and the third was not confluent with it. Alveoli are circular, and a diastema separates the eighth and ninth. The tenth and eleventh are enlarged relative to the anterior alveoli. The dentary symphysis extends to the level of the fifth dentary alveolus, or to a level immediately behind it. Lateral sulci between the seventh through ninth alveoli would have received opposing maxillary teeth.

The splenials do not meet at the midline. Its anteriormost extent is ventral to the slender Meckelian groove on the dentary at approximately the level of the sixth dentary alveolus (Fig. 6E). The splenial expands posteriorly and contributes to the medial alveolar borders beginning with the tenth dentary alveolus. It forms the anterodorsal border of the relatively large oval caudal foramen intermandibularis oralis, and there is no evidence for an anterior perforation.

One left (KNM BK II OLD 1960, Fig. 6A) and one right (KNM FLKNI) coronoid are preserved. Each is mediolaterally flat and communicates with the splenial anteriorly, angular ventrally, and (to a minor extent) the surangular dorsally. The actual outline is imperfectly preserved in both cases, but the KNM BK II mandible reveals a small medial foramen intermandibularis oralis. The dorsal ramus projects posteriorly for a short distance medial to the surangular, and its dorsal margin is oriented anteroposteriorly and does not slope anteriorly. The ventral ramus forms the ventromedial border of the adductor chamber. The coronoid appears to contribute to the caudal formen intermandibularis oralis, on the KNM specimen, but this most likely results from dorsoventral compression.

The angular has a broadly convex ventral surface. Its medial lamina forms the posteroventral and part of the dorsal margin of the caudal foramen intermandibularis oralis. Its lateral surface is smooth and unpitted where it forms the ventrolateral portion of the retroarticular process. Most preserved specimens (e.g. NNHM-OLD-1001, Fig. 2O) indicate a posterior ramus of the angular that extends roughly as far posteriorly as the surangular on the retroarticular process, but NHM R5893 suggests a truncated angular that terminates anterior to the surangular. Such a condition is highly unusual for any crocodylian, and in light of the consistently non-truncated angulars in other specimens, the NHM specimen is best viewed as aberrant.

The surangular (Fig. 6) bears a pair of anterior processes. The dorsalmost process extends anteriorly to the dentary toothrow, and the ventral process is anteroposteriorly shorter and dorsoventrally wider. Its contact with the dentary in lateral view is linear and intersects the external mandibular fenestra along its anterodorsal margin. The surangular forms the entire posterior margin of the fenestra (Fig. 6B); the holotype (Fig. 3O) suggests intersection of the surangular-angular suture at the posteriormost end of the mandibular fenestra, but this is because the slender process of the surangular that would extend to the ventral margin is broken off. The smooth dorsal surface extends laterally between the mandibular fenestra and glenoid fossa, forming a robust lateral shelf (Fig. 2S). It passes along the dorsolateral surface of the retroarticular process and extends all the way to the posterior tip. Dorsally, the surangular contributes to the lateral glenoid subfossa.

The descending ramus of the articular is triangular in cross-section, tapering to a rounded apex ventrally (Fig. 6). Its anterior surface is concave, and it bears a thin lamina on its lateral margin that passes along the medial surface of the surangular. A small foramen passes between the articular and surangular immediately ventral to this lamina. The glenoid fossa is comprised of two dorsal subfossae, and a sharply bowed angular-surangular suture passes through the lateral subfossa. The dorsal surface of the retroarticular process is also divided into two fossae separated by a low, broad anteroposterior ridge. A small foramen aereum pierces the articular at the anteromedial edge of the retroarticular process.

All associated teeth are conical and bear unserrated mesiodistal carinae.

Associated postcranial material is consistent with homologues in living species of *Crocodylus*. The atlas intercentrum is a wedge-shaped object with a dorsal concavity flooring the neural canal and prominent diapophyses (Fig. 3C). The axis centrum bears a robust hypapophysis behind the odontoid process, which in the holotype appears to have largely fused with the axial centrum (Fig. 3D), even though the neural arch had popped off along its sutural surface. Vertebrae are procoelous. The scapula has a relatively slender dorsal blade, a narrow deltoid crest, and mediolaterally wide body (Fig. 7A). The deltopectoral crest of the humerus was concave proximally (Figs. 3E, 7A). The lateral surface of the ilium is not visible on the holotype, but in posterior view it reveals a wasp-waisted posterior blade (Fig. 3G). The femur is sigmoid in shape and had shallow depressions for the caudofemoralis musculature on its ventral surface anterior and posterior to the fourth trochanter (Fig. 7D). Most osteoderms (presumably from the dorsal shield) are square in dorsal view and, in most cases, bear a robust dorsal keel (Fig. 3B); at least one (Fig. 7B) is oval in dorsal view, suggesting it is from the nuchal shield.

## Methods

*Crocodylus anthropophagus* was added to a matrix of 98 morphological characters and 34 ingroup taxa (Appendix S1). A maximum parsimony analysis was conducted using TNT 1.1 [44]. 100 random-seed heuristic searches were performed. *Borealosuchus sternbergii, Pristichampsus vorax*, and *Leidyosuchus canadensis* were used as sequential outgroups. Optimal trees were exported to PAUP 4.10b [45] to construct Adams consensus trees.

## Results

**Figure 8:**
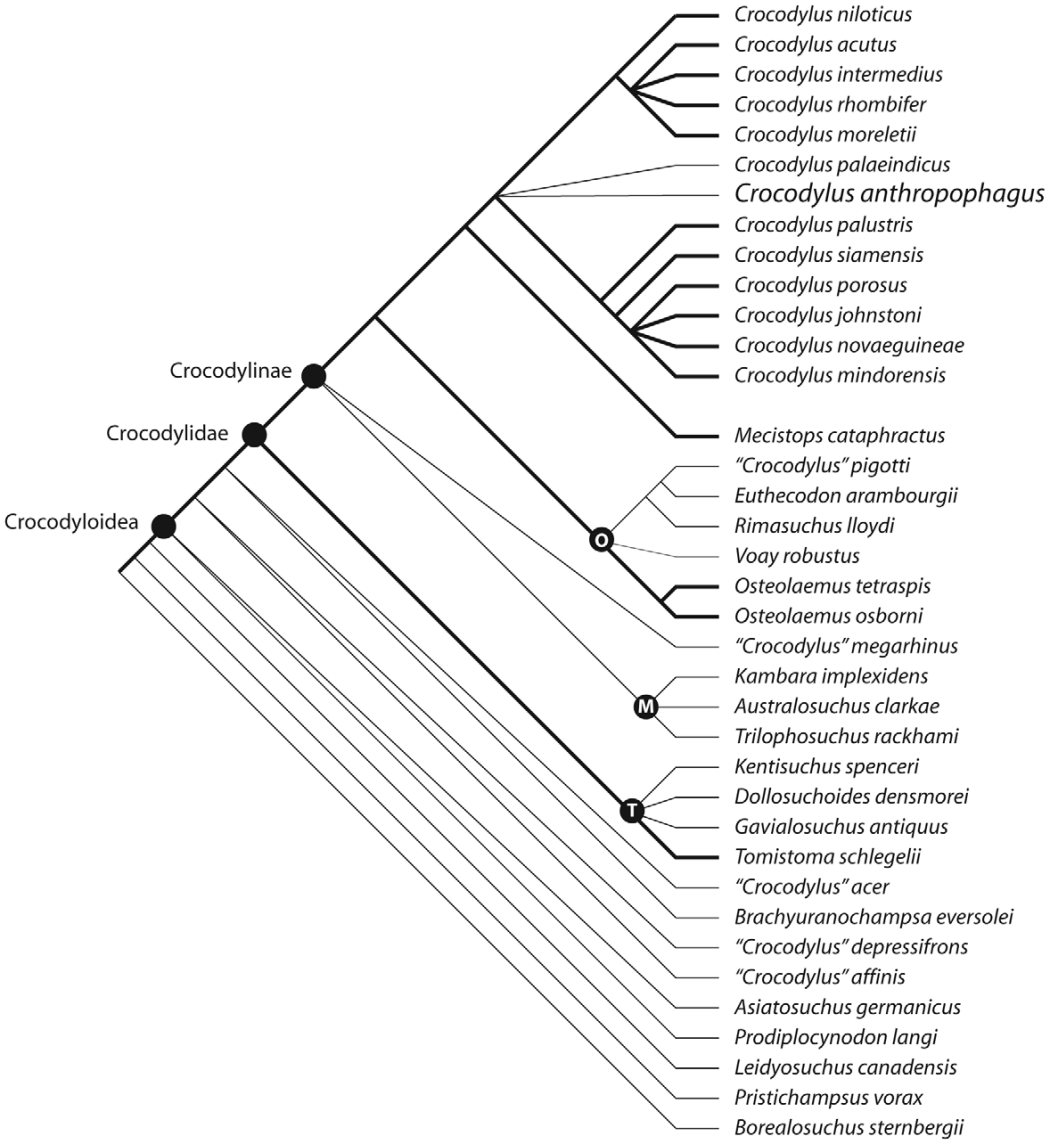
Phylogenetic relationships recovered by a maximum parsimony analysis of 98 morphological characters. Adams consensus of 426 equally optimal trees (length = 225, CI excluding uninformative characters = 0.493, RI = 0.717). Dashed lines indicate lost resolution in a strict consensus of the same trees. O = Osteolaeminae, M = Mekosuchinae, T = Tomistominae. Heavy branches indicate living lineages.

The heuristic searches recovered 426 equally optimal trees (length = 225, CI excluding uninformative characters = 0.493, RI = 0.717). Strict and Adams consensus trees of these results (Fig. 8) are broadly congruent with previous morphological analyses [23], [27], [46]. *Mecistops* is the closest relative of *Crocodylus*. Groups of Afro-Malagasy and Australasian forms – osteolaemines and mekosuchines, respectively – form subclades within Crocodylinae.

If the relationships among outgroup taxa are not constrained to reflect more inclusive analyses of Crocodylia (i.e. forcing *Leidyosuchus* to be closest to Crocodyloidea and *Borealosuchus sternbergii* as the basalmost outgroup), *Pristichampsus* is closer to Crocodyloidea and trees are one step shorter. Character sampling in this analysis was focused on variation among crocodyloids. Most of the characters relevant to relationships among non-crocodyloid lineages were not included.

*Crocodylus* is less resolved than in previous morphological analyses. This reflects incompleteness in two extinct species - *Crocodylus anthropophagus* and *C. palaeindicus*. *Crocodylus anthropophagus* assumes seven positions in the optimal trees – closely related to *C. niloticus*, *C. rhombifer*, *C. palaeindicus*, *C. siamensis*, the Neotropical clade, the Afro-Neotropical group, or the Indopacific group. Adams consensus trees (Fig. 8) restore the close relationship between the Neotropical species and *C. niloticus* supported by morphological [23] and molecular [24], [25] evidence.

Placements of *C. anthropophagus* within the Indopacific or Neotropical clades (other than as a close relative to *C. rhombifer* or *C. siamensis*) increase tree length by only one step. None of the most parsimonious placements has bootstrap support exceeding 50%. Hence, although the data analyzed here support placement of *C. anthropophagus* close to (if not within) *Crocodylus*, we are unable to pinpoint its relationships more precisely.

## Discussion

### Phylogenetic Relationships

A close relationship between *Crocodylus anthropophagus* and extant *Crocodylus* is supported by several unambiguous character states. In all crocodylians, the pharynx pneumatizes the braincase through three small openings (the Eustachian foramina) between the basioccipital and basisphenoid on the occipital plate [47], [48]. Osteolaemines (including *Rimasuchus*) and *Mecistops* share the ancestral condition in which the lateral foramina are located dorsal to the median foramen. In *Crocodylus* the lateral foramina are located ventrally and almost in line with the median foramen [23]. This coincides with a decrease in the dorsoventral depth of the pterygoid ventral to the median Eustachian foramen, which in turn limits the exposure of the basisphenoid ventral to the basioccipital on the posteroventral surface of the skull. This is the condition found in *C. anthropophagus* (Fig. 5C).

The medial wall of the caviconchal recess – a large pneumatic feature in the maxilla dorsomedial to the toothrow – is perforated with a linear array of blind pits in *C. anthropophagus* (Fig. 4H). This is a derived feature found only in *Crocodylus* [23], [49]. The condition in *Rimasuchus* is unknown, but they are absent from *Osteolaemus*, *“Crocodylus” pigotti*, and *Voay* [27] (pers. obs.).

An isolated ilium associated with the *C. anthropophagus* holotype reveals a deeply concave dorsal and ventral margin to the posterior blade, resulting in the “wasp-waisted” condition found in *Crocodylus* but absent from other crocodyloids (Fig. 3G) [23]. The ilium of *R. lloydi* is unknown, but the posterior blade of *Voay* lacks substantial notching [27].

Derived states typically found in osteolaemines are absent from *C. anthropophagus*. The quadrate-squamosal suture follows the sulcus between the paroccipital process and anterior quadrate ramus, and the squamosal does not lap over the dorsal surface of the ramus. The surface of the fused pterygoids anterior to the internal choana is elevated, but the elevation apparently does not surround the chaoanal aperture as it does in osteolaemines, and there is no choanal neck. Trees supporting a close relationship between *C. anthropophagus* and *R. lloydi* are minimally seven steps longer than optimal.

Cranial ornamentation features that diagnose *C. anthropophagus* are elaborations of features found among most derived crocodyloids. The orbital rim is upturned in all extant *Crocodylus*, but discrete knobs on the prefrontal are either absent or weakly developed, and there is usually a discontinuity between the upturned orbital margin and any dorsal reflection of the lateral skull table margin. An anteroposterior crest is usually found on the dorsal surface of the lacrimal in crocodylids, though it is especially well-developed in most Indo-Pacific species of *Crocodylus* and some extinct osteolaemines. But in these, the crest takes the form of a long continuous ridge, not the discrete knobs seen in *C. anthropophagus*.

These ornamental features are sufficient to distinguish *C. anthropophagus* from most other Neogene crocodylines. *Crocodylus checchiai* from the Miocene of Libya [7], [11], [50], *Crocodylus gariepensis* from the Miocene of Namibia [4], and Mio-Pliocene fossils referred to *Crocodylus* from Italy [46], [51], [52], the Manonga Valley of Tanzania [53](pers. obs.), and Abu Dhabi [54] uniformly lack squamosal horns and discrete prefrontal knobs. The squamosals of large crocodiles from the Late Miocene and Pliocene Lothagam and Koobi Fora localities referred in the past to *Rimasuchus lloydi* [2], [17], however, are dorsally inflated. Although not to the degree seen in *C. anthropophagus*, this contrasts the Kenyan skulls with *R. lloydi* from the type locality [55] (pers. obs.), all of which have flat skull tables.

**Figure 9:**
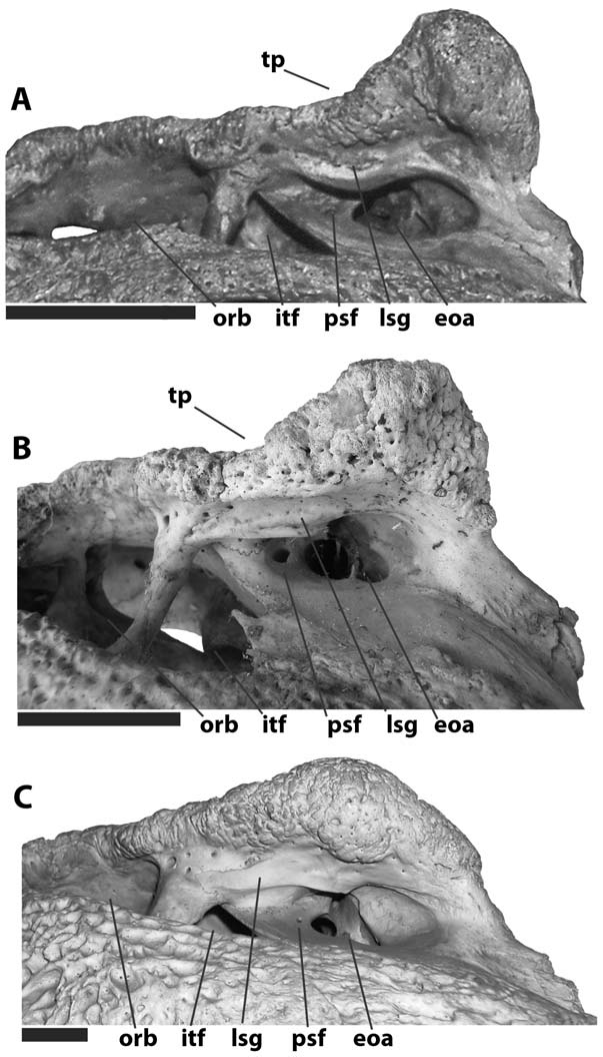
Squamosal horns of living and extinct crocodylines, left lateral view. A, AMNH 3101, *Voay robustus* (right lateral view, photo inverted). B, PNCZ unnumbered, *Crocodylus rhombifer*. C, NHM 94.6.5.53, *C. niloticus*. Scale = 5 cm.

Posterodorsal squamosal horns characterize the Cuban (Fig. 9B) and Siamese crocodiles [43], [56]. Like *C. anthropophagus*, the horns of these species are sharply demarcated in both posterior and lateral view, at least in larger individuals. It is because of these structures that trees linking *C. anthropophagus* to *C. rhombifer* or *C. siamensis* are among the optimal arrangements. Nevertheless, *C. anthropophagus* can be readily distinguished from either living species; *C. rhombifer*, like other Neotropical species, has a prominent dorsal boss on the rostrum not present in *C. anthropophagus*, and *C. anthropophagus* lacks the prominent long preorbital crest found in most Indopacific species of *Crocodylus* (including *C. siamensis*) and the midline crest on the frontal diagnostic of *C. siamensis* [23], [57], [58].

Although not as prominent, dorsally expanded squamosals are sometimes found in very large specimens of most other living species of *Crocodylus*, including *C. niloticus* (Fig. 9C). The horns of *C. anthropophagus* are more prominent and have more acute dorsal tips than these structures, and in lateral view, there is an abrupt transition from the dorsal surface of the postorbital (which is parallel to the coronal plane) and the upturned squamosal horn. This is most apparent on the KNM FLKNI right squamosal (Fig. 4N), though this is case as well for the holotype (Fig. 1H). Although true for *Voay* (Fig. 9A) and large *C. rhombifer* (Fig. 9B) and *C. siamensis*, this is unlike the condition in other species of *Crocodylus*; when present, the dorsal expansion arises more gradually behind the postorbital bar (Fig. 9C).

A few extinct crocodylians also bear squamosal horns similar to those of *C. anthropophagus*, including the osteolaemine *Voay robustus* from the Quaternary of Madagascar (Fig. 9A)[59], [60], [61]. Indeed, squamosal horns of *V. robustus* and *C. anthropophagus* are similar enough that isolated elements may not be assignable to either species. Skeletal morphology strongly supports a close relationship between *Voay* and *Osteolaemus*, and squamosal horns are best viewed as independently derived features in *Voay* and *C. anthropophagus*.

Another crocodylid with squamosal horns is *Aldabrachampsus dilophus* from the Quaternary of Aldabra Atoll [14]. *Aldabrachampsus* is incompletely known and its phylogenetic relationships are unclear, but its horns differ from those of both *Voay* and *C. anthropophagus*; they are broad and oblique in lateral view, with an apex anterodorsal rather than dorsal to the otic aperture. Moreover, known material of *Aldabrachampsus* suggests a very small animal (∼2m) at maturity, and the holotype of *C. anthropophagus* is from a substantially larger animal.

**Figure 10:**
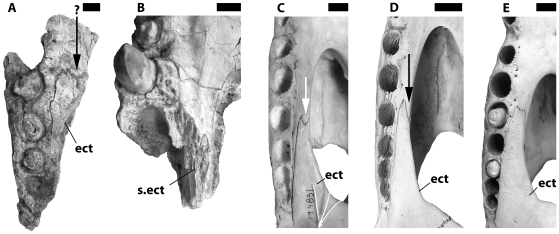
Variation in the morphology of the maxillary ramus of the ectopterygoid. All images from right side of skull in ventral view. A, NHM R.5893, *Crocodylus anthropophagus*, posterior end of right maxilla and partial maxillary ramus of ectopterygoid. B, KNM FLKNI, *C. anthropophagus*, partial right maxilla; articulation surface for ectopterygoid is preserved. C, USNM 194831, *C. niloticus*. D, USNM 248848, *C. niloticus*. E, FMNH 17157, *C. niloticus*. Arrow indicates cleft in maxillary ramus of ectopterygoid; questionably present in A, on medial margin of suborbital fenestra in D. Scale = 1 cm.

One character might suggest monophyly of extant *Crocodylus* to the exclusion of *C. anthropophagus* – a cleft in the maxillary ramus of the ectopterygoid. Preserved ectopterygoids and maxillae of *C. anthropophagus* suggest an unforked maxillary ramus that tapers anteriorly (Fig. 10A,B), the condition found in all other crocodylians. Cleft maxillary rami (Fig. 10C) are only seen in *Crocodylus*, and it was coded as present in all species in previous analyses [23]. If these codings are applied to the present analysis, *C. anthropophagus* is unambiguously outside (albeit close to) crown *Crocodylus*.

But further examination of *Crocodylus* skulls indicates variability in living species – the cleft is not apparent in some individuals (Fig. 10E), and it lies right on the margin of the suborbital fenestra in others, making the medial tine of the fork difficult to see in ventral view (Fig. 10D). This character (63) was thus recoded as polymorphic in all living species, causing alternative placements of *C. anthropophagus* to become no less parsimonious. Variability was not observed in *C. palaeindicus*, and it remains coded as monomorphic for this trait, but fewer specimens are available and a larger sample may eventually reveal polymorphism.

Even fewer specimens of *C. anthropophagus* preserve the relevant parts of the skull, and our confidence that the species uniformly lacked the cleft is less than robust. Moreover, the partial right ectopterygoid of NHM R5893 (Fig. 10A) bears a slight concavity on its anterior tip. We have interpreted this structure as unforked, but one could argue for the forked condition. Recoding *C. anthropophagus* as polymorphic has no impact on the results of the parsimony analysis.

### *Crocodylus anthropophagus* and *Crocodylus niloticus*

We have no complete skulls for *C. anthropophagus* and, thus, no solid grasp of the shape of the snout, but compared with *C. niloticus*, the premaxillae and maxillae indicate a comparatively deeper snout with a more highly vaulted palate; a relatively shorter prenarial rostrum (Fig. 1A,E); a naris with more anterior orientation; and more prominent crests along the margins of the orbit and skull table. *Crocodylus niloticus* lacks the prominent crest along the maxillonasal suture seen in *C. anthropophagus*. Although squamosal horns sometimes appear in *C. niloticus*, they are rarely (if ever) as clearly demarcated from the dorsal surface of the skull table as in *C. anthropophagus*, and they are neither as prominent nor as sharply angled dorsally (Fig. 9C). Moreover, they appear in all observed squamosals of *C. anthropophagus*, including some from animals probably between 2 and 3 m in length, which suggests regularity in expression absent from *C. niloticus*, in whom upturned squamosals are only found in some very large individuals (>3 m).

Nevertheless, differentiation of isolated fragments of *C. anthropophagus* and *C. niloticus* may not always be possible, and this bears on interpretations of the Plio-Pleistocene crocodylian record in Africa. Fossils as old as the Miocene have been referred to *C. niloticus* [2], [17]; whether these are conspecific with *C. niloticus* (or even assignable to *Crocodylus*) is doubtful [62], but some geologically younger specimens (e.g. specimens forming the basis of *C. niloticus kaisensis* from the Pleistocene of Uganda [63]) are more consistent with the living species than with *C. anthropophagus* (pers. obs.). At least two similar species of *Crocodylus* may have been present in East Africa during the Late Pliocene and Pleistocene, and in the absence of diagnostic features permitting precise identification [64], [65], referral of fragmentary remains to the species level may not be advisable.

Preliminary analyses of the phylogeny of Neogene African crocodiles suggested that *Crocodylus* might be a comparatively recent immigrant into Africa and not a native lineage [30]. This was based on incomplete taxonomic sampling, and more recent work including a wider range of Mio-Pliocene forms suggests a more complicated phylogenetic and biogeographic history for the group in the region [62], but assuming *Crocodylus* was absent from Africa in the Early and Middle Miocene, the presence of two species in at least the early Pleistocene, if not the Pliocene, suggests either multiple dispersal events or dispersal early enough to have radiated by the Pleistocene. Further analysis of Late Miocene and Pliocene fossils from the region is needed to test these scenarios, but regardless, crocodiles appear to have remained cryptically speciose in Africa beyond their peak of diversity in the Miocene.

That the features distinguishing *C. anthropophagus* from *C. niloticus* are dominated by gradational differences raises the general problem of how we recognize species in the fossil record. It is possible that Olduvai Gorge crocodile is an extinct regional variant of the Nile crocodile and not a discrete species. Molecular evidence reveals considerable genetic variation between populations of *C. niloticus* [18], [66]. However, biogeographic variation in *C. niloticus* morphology is expressed almost entirely in scalation [67]. Different living populations of *C. niloticus* may ultimately be distinguishable osteologically, but the differences will be subtle and most apparent from morphometric rather than qualitative approaches. Qualitatively, the fossil Olduvai crocodile lies outside the range of osteological variation for *C. niloticus*, both within and between populations. Cranially, the Olduvai form can be consistently distinguished from *C. niloticus*, and we cannot at present conclude that one is phylogenetically closely related to the other, even if biogeography strongly suggests such a relationship.

### Paleoecology

Fossil bones of at least two hominid individuals from Olduvai Gorge bear tooth marks characteristic of crocodile feeding [37]. These marks are similar to those produced by mammalian carnivores, except that they are bisected by the carinae of newly erupted to moderately worn crocodile teeth [68]. Both tooth-marked specimens are from the same Tuff IB-IC interval as NNHM-OLD-1001, and were found by the Leakeys (L. Leakey, 1959; L. Leakey et al, 1964, M. Leakey, 1971) at two sites within 100 m of the collection site for NNHM-OLD-1001. Both hominid sites contain concentrations of vertebrate fossils and Oldowan stone artifacts. The FLK NN Level 3 site yielded the tooth-marked Olduvai Hominid (OH) 8 foot, a paratype of *H. habilis* found in the same assemblage as the species holotype. *In situ* elements of the *C. anthropohagus* holotype are essentially contemporaneous with OH 8. The FLK Level 22 site yielded the tooth-marked OH 35 tibia and fibula, probably of *H. habilis* [69], from the same assemblage as the holotype of *A. boisei*. Both OH 8 and OH 35 are from the left leg of a juvenile or adult [69], and have been argued to represent a single individual on the basis of their close articulation [70], despite deriving from different sites. Recent stratigraphic correlations of the sites show that these formed on two allochronous land surfaces [71]. Curiously, the tooth mark patterning on both specimens indicates that each hominid individual lost its left foot to crocodiles during or shortly after capture, or when being scavenged [37].

The FLK 22 and FLK NN 3 sites formed in close proximity (<50 m) to wetland settings from which crocodile body and trace fossils are documented [35], [71]. FLK 22 formed on a topohigh adjacent to a freshwater marshland, and FLK NN 3 formed on the base of a shallow floodplain channel. NNHM-OLD-1001 likely derives from the floodplain deposits adjacent to this channel. The tooth-marked hominids died and were fed on by crocodiles at either the wetlands or the sites at which their remains were found.

Predation risk from crocodiles likely impacted the foraging and land use behavior of hominids at Olduvai and at other tropical and sub-tropical near-wetland sites. Crocodiles were the largest predators encountered by hominids and are commonly found in the lake and river basins that also preserve fossil hominids in East Africa and elsewhere [2], [72], [73], [74], [75], [76], [77]. They inhabit settings that afforded hominids potable water and rich food sources, in particular rootstock from marsh plants and scavengeable larger mammal carcasses [78]. Given the relatively small body sizes of fossil hominids pre-dating *H. erectus* (e.g., *H. habilis* at <1 m tall and <40 kg body weight; *P. boisei* at <1.4 m tall, 80 kg body weight), crocodile feeding traces would likely have been inflicted by younger small- to medium-sized crocodiles, as estimated from tooth mark size for OH 8 and 35 [37]. Larger crocodiles would be capable of consuming hominids completely, leaving no trace. Crocodiles may have been common hominid predators, and as such should be considered in discussions of the ecological context of human origins.

## Supporting Information

Appendix S1 List of characters and character matrix used in this analysis.
(0.06 MB DOC)
