# Supplementary material for: A New Horned Crocodile from the Plio-Pleistocene Hominid Sites at Olduvai Gorge, Tanzania
Source: PLoS One. 2010 Feb 24;5(2):e9333. doi: 10.1371/journal.pone.0009333 (PMC2827537; doi:10.1371/journal.pone.0009333)
Supplement: Appendix S1 — List of characters and character matrix used in this analysis. (0.06 MB DOC) [file pone.0009333.s001.doc]

**Appendix S1: List of characters and character matrix used in this analysis.** Number in parentheses next to character number in the character list indicates corresponding character number in earlier versions of this matrix (e.g., Brochu, 1999, 2004, 2007).

1 (1). Ventral tubercle of proatlas more than one-half (0) or no more than one half (1) the width of the dorsal crest.

2 (2). Fused proatlas boomerang-shaped (0), strap-shaped (1), or massive and block-shaped (2).

3 (10). Proatlas with prominent anterior process (0) or lacks anterior process (1).

4 (14). Dorsal margin of atlantal rib generally smooth with modest dorsal process (0) or with prominent process (1).

5 (20). Axial rib tuberculum wide, with broad dorsal tip (0) or narrow, with acute dorsal tip (1).

6 (11). Anterior half of axis neural spine oriented horizontally (0) or slopes anteriorly (1).

7 (12). Axis neural spine crested (0) or not crested (1).

8 (3). Posterior half of axis neural spine wide (0) or narrow (1).

9 (7). Hypapophyseal keels present on eleventh vertebra behind atlas (0), twelfth vertebra behind atlas (1), or tenth vertebra behind atlas (2).

10 (8). Third cervical vertebra (first postaxial) with prominent hypapophysis (0) or lacks prominent hypapophysis (1). (Adapted from Norell, 1989, character 12; Norell and Clark, 1990, character 11; Clark, 1994, character 91.)

11 (9). Neural spine on third cervical long, dorsal tip at least half the length of the centrum without the cotyle (0) or short, dorsal tip acute and less than half the length of the centrum without the cotyle (1).

12 (13). Anterior sacral rib capitulum projects far anteriorly of tuberculum and is broadly visible in dorsal view (0), or anterior margins of tuberculum and capitulum nearly in same plane, and capitulum largely obscured dorsally (1).

13 (22). Scapular blade flares dorsally at maturity (0) or sides of scapular blade subparallel; minimal dorsal flare at maturity (1). (Adapted from Benton and Clark, 1988.)

14 (25). Scapulocoracoid facet anterior to glenoid fossa uniformly narrow (0) or broad immediately anterior to glenoid fossa, and tapering anteriorly (1).

15 (26). Proximal edge of deltopectoral crest emerges smoothly from proximal end of humerus and is not obviously concave (0) or emerges abruptly from proximal end of humerus and is obviously concave (1).

16 (27). Olecranon process of ulna narrow and subangular (0) or wide and rounded (1).

17 (30). Interclavicle flat along length, without dorsoventral flexure (0) or with moderate dorsoventral flexure (1) or with severe dorsoventral flexure (2).

18 (34). Iliac anterior process prominent (0) or virtually absent (1). (Adapted from Benton and Clark, 1988; Clark, 1994, character 84; although the transformation recorded here is different.)

19 (28). Dorsal margin of iliac blade rounded with smooth border (0) or rounded, with modest dorsal indentation (1) or rounded, with strong dorsal indentation (“wasp-waisted;” 2) or narrow, with dorsal indentation (3) or rounded with smooth border; posterior tip of blade very deep (4).

20 (32). Supraacetabular crest narrow (0) or broad (1).

21 (33). Limb bones relatively robust, and hindlimb much longer than forelimb at maturity (0) or limb bones very long and slender (1).

22 (35). Dorsal osteoderms not keeled (0) or keeled (1). (Adapted from Buscalioni et al., 1992, character 22.)

23 (36). Dorsal midline osteoderms rectangular (0) or nearly square (1). ) Adapted from Norell and Clark, 1990, character 16; Clark, 1994, character 95.)

24 (38). Nuchal shield grades continuously into dorsal shield (0) or differentiated from dorsal shield; four nuchal osteoderms (1) or differentiated from dorsal shield; six nuchal osteoderms with four central and two lateral (2) or differentiated from dorsal shield; eight nuchal osteoderms in two parallel rows (3).

25 (39). Ventral armor absent (0) or single ventral osteoderms (1) or paired ventral ossifications that suture together (2). (Adapted from Buscalioni et al., 1992, character 21.)

26 (40). Anterior margin of dorsal midline osteoderms with anterior process (0) or smooth, without process (1). (Adapted from Norell and Clark, 1990, character 13; Clark, 1994, character 96.)

27 (52). Alveoli for dentary teeth 3 and 4 nearly same size and confluent (0) or fourth alveolus larger than third, and alveoli are separated (1).

28 (166). Dentary symphysis extends to fourth or fifth alveolus (0) or sixth through eighth alveolus (1) or behind eighth alveolus (2.)

29 (68). Dentary gently curved (0), deeply curved (1), or linear (2) between fourth and tenth alveoli.

30 (167). Largest dentary alveolus immediately caudal to fourth is (0) 13 or 14, (1) 13 or 14 and a series behind it, (2) 11 or 12, or (3) no differentiation, or (4) behind 14.

31 (41). Splenial with anterior perforation for mandibular ramus of cranial nerve V (0) or lacks anterior perforation for mandibular ramus of cranial nerve V (1). (Adapted in part from from Norell, 1988, character 15 and 1989, character 8.)

32 (43). Splenial participates in mandibular symphysis; splenial symphysis adjacent to no more than five dentary alveoli (0) or splenial excluded from mandibular symphysis; anterior tip of splenial passes ventral to Meckelian groove (1) or splenial excluded from mandibular symphysis; anterior tip of splenial passes dorsal to Meckelian groove (2) or deep splenial symphysis, longer than five dentary alveoli; splenial forms wide “V” within symphysis (3) or deep splenial symphysis, longer than five dentary alveoli; splenial constricted within symphysis and forms narrow “V” (4). (Adapted from Clark, 1994, character 77.)

33 (54). Superior edge of coronoid slopes strongly anteriorly (0) or almost horizontal (1).

34 (47). Angular-surangular suture contacts external mandibular fenestra at posterior angle at maturity (0) or passes broadly along ventral margin of external mandibular fenestra late in ontogeny (1). (Adapted from Norell, 1988, character 40.)

35 (61). Surangular with spur bordering the dentary toothrow lingually for at least one alveolus length (0) or lacking such spur (1).

36 (106). Surangular continues to dorsal tip of lateral wall of glenoid fossa (0) or truncated and not continuing dorsally (1).

37 (44). Articular-surangular suture simple (0) or articular bears anterior lamina dorsal to lingual foramen (1) or articular bears anterior lamina ventral to lingual foramen (2) or bears laminae above and below foramen (3.)

38 (45). Lingual foramen for articular artery and alveolar nerve perforates surangular entirely (0) or perforates surangular/angular suture (1).

39 (49). Foramen aerum at extreme lingual margin of retroarticular process (0) or set in from margin of retroarticular process (1). (Adapted from Norell, 1988, character 16.)

40 (162). Surangular-articular suture oriented anteroposteriorly (0) or bowed strongly laterally (1) within glenoid fossa.

41 (60). Sulcus between articular and surangular (0) or articular flush against surangular (1).

42 (57). Dorsal projection of hyoid cornu flat (0) or rodlike (1).

43 (166). Teeth and alveoli of maxilla and/or dentary circular in cross-section (0), or posterior teeth laterally compressed (1), or all teeth compressed (2.)

44 (79). Naris projects anterodorsally (0) or dorsally (1).

45 (95). External naris bisected by nasals (0) or nasals contact external naris, but do not bisect it (1) or nasals excluded, at least externally, from naris; nasals and premaxillae still in contact (2) or nasals and premaxillae not in contact (3). (Adapted from Norell, 1988, character 3; Clark, 1994, characters 13 and 14.)

46 (97). Premaxilla has five teeth (0) or four teeth (1) early in posthatching ontogeny. (Norell, 1988, character 17.)

47 (153). Incisive foramen completely situated far from premaxillary toothrow, at the level of the second or third alveolus (0) or abuts premaxillary toothrow (1) or projects between first premaxillary teeth (2).

48 (145). Dorsal premaxillary processes short, not extending beyond third maxillary alveolus (0) or long, extending beyond third maxillary alveolus (1).

49 (78). All dentary teeth occlude lingual to maxillary teeth (0) or occlusion pit between 7th and 8th maxillary teeth; all other dentary teeth occlude lingally (1) or dentary teeth occlude in line with maxillary toothrow (2). (Adapted from Norell, 1988, character 5; Willis, 1993, character 1.)

50 (89). Largest maxillary alveolus is #3 (0), #5 (1), #4 (2), #4 and #5 are same size (3), #6 (4), or maxillary teeth homodont (5), or maxillary alveoli increase in diameter posteriorly toward penultimate alveolus (6). (Adapted from Norell, 1988, character 1.)

51 (135). Maxillary toothrow curved medially or linear (0) or curves laterally broadly (1) posterior to first six maxillary alveoli. (Adapted from Clark, 1994, character 79.)

52 (101). Dorsal surface of rostrum curves smoothly (0) or bears medial dorsal boss (1).

53 (144). Preorbital ridges absent or very modest (0) or very prominent (1) at maturity.

54 (126). Vomer entirely obscured by maxillae and palatines (0) or exposed on palate between palatines (1).

55 (148). Surface of maxilla within narial canal imperforate (0) or with a linear array of pits (1.)

56 (120). Medial jugal foramen small (0) or very large (1).

57 (111). Maxillary foramen for palatine ramus of cranial nerve V small or not present (0) or very large (1).

58 (91). Ectopterygoid abuts maxillary tooth row (0) or maxilla broadly separates ectopterygoid from maxillary tooth row (1). (Norell, 1988, character 19.)

59 (136). Medial process of prefrontal pillar expanded dorsoventrally (0) or anteroposteriorly (1).

60 (138). Medial process of prefrontal pillar wide (0) or constricted (1) at base.

61 (105). Maxilla has linear medial margin adjacent to suborbital fenestra (0) or bears broad shelf extending into fenestra, making lateral margin concave (1.)

62 (108). Anterior face of palatine process rounded or pointed anteriorly (0) or notched anteriorly (1).

63 (109). Anterior ectopterygoid process tapers to a point (0) or forked (1).

64 (110). Palatine process extends (0) or does not extend (1) significantly beyond anterior end of suborbital fenestra. (Adapted from Willis, 1993, character 2.)

65 (118). Palatine process generally broad anteriorly (0) or in form of thin wedge (1).

66 (94). Lateral edges of palatines smooth anteriorly (0) or with lateral process projecting from palatines into suborbital fenestrae (1).

67 (85). Palatine-pterygoid suture nearly at (0) or far from (1) posterior angle of suborbital fenestra.

68 (88). Pterygoid ramus of ectopterygoid straight, posterolateral margin of suborbital fenestra linear (0) or ramus bowed, posterolateral margin of fenestra concave (1.)

69 (73). Pterygoid surface lateral and anterior to internal choana flush with choanal margin (0) or pushed inward anterolateral to choanal aperture (1) or pushed inward around choana to form “neck” surrounding aperture (2) or everted from flat surface to form “neck” surrounding aperture (3).

70 (152). Internal choana not septate (0) or with septum that remains recessed within choana (1) or with septum that projects out of choana (2).

71 (93). Lacrimal makes broad contact with nasal; no posterior process of maxilla (0) or maxilla with posterior process within lacrimal (1) or maxilla with posterior process between lacrimal and prefrontal (2).

72 (70). Postorbital bar massive (0) or slender (1). (Norell, 1989, character 3.)

73 (103). Margin of orbit flush with skull surface (0) or dorsal edges of orbits upturned (1) or orbital margin telescoped (2).

74 (96). Palpebral forms from single ossification (0) or from multiple ossifications (1). (Adapted from Norell, 1988, character 8; Clark, 1994, character 65.)

75 (114). Quadratojugal spine low, near posterior angle of infratemporal fenestra (0) or high, between posterior and superior angles of infratemporal fenestra (1).

76 (75). Quadratojugal forms posterior angle of infratemporal fenestra (0) or jugal forms posterior angle of infratemporal fenestra (1) or quadratojugal-jugal suture lies at posterior angle of infratemporal fenestra (2). (Adapted from Norell, 1989, character 10.)

77 (76). Postorbital neither contacts quadrate nor quadratojugal medially (0) or contacts quadratojugal, but not quadrate, medially (1) or contacts quadrate and quadratojugal at dorsal angle of infratemporal fenestra (2) or contacts quadratojugal with significant descending process (3).

78 (83). Quadratojugal bears long anterior process along lower temporal bar (0) or bears modest process, or none at all, along lower temporal bar (1).

79 (80). Quadratojugal extends to superior angle of infratemporal fenestra (0) or does not extend to superior angle of infratemporal fenestra; quadrate participates in fenestra (1). (Adapted from Buscalioni et al., 1992, character 6.)

80 (84). Dorsal and ventral rims of squamosal groove for external ear valve musculature parallel (0) or squamosal groove flares anteriorly (1).

81 (132). Squamosal-quadrate suture extends dorsally along posterior margin of external auditory meatus (0) or extends only to posteroventral corner of external auditory meatus (1).

82 (102). Posterior margin of otic aperture smooth (0) or bowed (1).

83 (81). Frontoparietal suture deeply within supratemporal fenestra; frontal prevents broad contact between postorbital and parietal (0) or suture makes modest entry into supratemporal fenestra at maturity; postorbital and parietal in broad contact (1) or suture on skull table entirely (2).

84 (86). Frontoparietal suture concavoconvex (0) or linear (1) between supratemporal fenestrae.

85 (87). Supratemporal fenestra with fossa; dermal bones of skull roof do not overhang rim at maturity (0) or dermal bones of skull roof overhang rim of supratemporal fenestra near maturity (1) or supratemporal fenestra closes during ontogeny (2). (Adapted from Norell, 1988, character 9.)

86 . Posterolateral margin of squamosal horizontal or nearly so (0) or upturned to form a discrete “horn” (1.)

87 (150). Squamosal does not extend (0) or extends (1) ventrolaterally to lateral extent of paraoccipital process.

88 (82). Supraoccipital exposure on dorsal skull table small (0), absent (1), large (2), or large such that parietal is excluded from posterior edge of table (3). (Norell, 1988, character 11.)

89 (122). Sulcus on anterior braincase wall lateral to basisphenoid rostrum (0) or braincase wall lateral to basisphenoid rostrum smooth; no sulcus (1).

90 (129). Basisphenoid not exposed extensively (0) or exposed extensively (1) on braincase wall anterior to trigeminal foramen (Adapted from Norell, 1989, character 5.)

91 (74). Extensive exposure of prootic on external braincase wall (0) or prootic largely obscured by quadrate and laterosphenoid externally (1). (Adapted from Norell, 1989, character 5.)

92 (127). Significant ventral quadrate process on lateral braincase wall (0) or quadrate-pterygoid suture linear from basisphenoid exposure to trigeminal foramen (1).

93 (128). Lateral carotid foramen opens lateral (0) or dorsal (1) to basisphenoid at maturity.

94 (98). Posterior pterygoid processes tall and prominent (0) or small and project posteroventrally (1) or small and project posteriorly (2).

95 (119). Basisphenoid not broadly exposed ventral to basioccipital at maturity; pterygoid short ventral to median eustachian opening (0) or basisphenoid exposed as broad sheet ventral to basioccipital at maturity; pterygoid tall ventral to median eustachian opening (1).

96 (147). Lateral eustachian canals open dorsal (0) or lateral (1) to medial eustachian canal. (Adapted from Norell, 1988, character 46.)

97 . quadrate foramen aereum is small (0), comparatively large (1), or absent (2) at maturity.

98 (112). Quadrate with small, ventrally-reflected medial hemicondyle (0) or with small medial hemicondyle; dorsal notch for foramen aerum (1) or with prominent dorsal projection between hemicondyles (2) or with expanded medial hemicondyle (3).

Borealosuchus sternbergii

00000110?10000000000100??001020000000000000020001310000100??0000011101010?001000000000000000001000

Pristichampsus vorax

?????01001??0001?010010??1110?00?0?001?0??2010?0030010?000??0000001001011??0100010000000????101002

Leidyosuchus canadensis

???????????0001??10?011?11010?0000?000101?0010000300000001001000011101010?101000010000000000001001

Mecistops cataphractus

10?1100000011111212001110111041111011101?101201021000001001100001011010110010110012000001111110003

Crocodylus niloticus

10101101001111112120011201100211111111011101101021000011001100{01}00011011110010110012000001111110103

Crocodylus porosus

11101001101110112120011201100211111111011101101021001011001100{01}00011010110010110012000001111110103

Crocodylus rhombifer

00101101001111112110011201100211111111011101101021010011001100{01}00011011110010110012001001111110103

Crocodylus acutus

00101101001111112120011201100211111111011101101021010011001100{01}00011011110010110012000001111110103

Crocodylus palustris

10101001011111112121011201100211111111011101101021001011001100{01}00011010110010110012000001111110103

Crocodylus siamensis

11101111000111012120011201100211111111011101101021001011001100{01}00011010110010110012001001111110103

Crocodylus intermedius

00101101001111112120011201110211111111011101101021010011001100{01}00011010110010110012000001111110103

Crocodylus johnstoni

111010011011101121200112011?0211111111011101101021001011001100{01}01011010110010110012000001111110103

Crocodylus mindorensis

11101001101110112120011201100211111111011101101021001011001100{01}00011010110010110012000001111110103

Crocodylus novaeguineae

11101001101110112120011201100211111111011101101021001011001100{01}00011010110010100012000001101110103

Crocodylus moreletii

00101101001111112120011201100211111111011101101021010011001100{01}00011011110010110012000001111110103

Crocodylus palaeindicus

???????????1?????1????????10021111111?011?0110?0210000?100110010000101011?010110012000010111110103

Crocodylus anthropophagus

????????????111??120011??11?021111?111011?00101021000?1100110?0???1?0??11??1?100011001001?1?110103

Euthecodon arambourgii

???????????????????????????????0???????1??0020?1250010?10011000000???0?11?11011001210010????1??0?3

Osteolaemus tetraspis

??101001100111112111011111100211111011011111011021001001001110010111110111010100012110101111111003

Osteolaemus osborni

??101001100111112111011111100211101011011111111021001001001110010010110111010100012110101111111003

Voay robustus

???????????1???1?1110????1110211101111111?0110?02100100100110001011111011?01011001201111?111110003

Rimasuchus lloydi

???????????????????????????????1???????1??0110?0210010?1001100000????1111?01011001200010111?11?0?3

“Crocodylus” pigotti

?????00??001?111?????10??01102?1???111?1??0010?02100100100??0010101110?11??1?1?001210010??1?10?003

“Crocodylus” megarhinus

??????????0???????????????110211?010??011?0110?023000001?0??0000001101211?02?110012?000?110?110003

Australosuchus clarkae

?????????0????1?????010??1110?11?01011011?0110112100000100??000000???1011?0201101120000011??1?00?1

Kambara implexidens

??????????????1??110010??1110211?01011011?0110112100000100?10000001001011?020110012000001111110001

Trilophosuchus rackhami

??????????????????????????????????????????0?????2?0?00?100??1?000001???11?1201101121000211111??0?1

Tomistoma schlegelii

021010010011011111100103011?2?14010000010001201121000101001100010010011110001100012100001101110003

Gavialosuchus antiquus

0200?001?001011??11?000??11?2??41??031?1?0012011210000?100??0000101?01101??011?101200001??0?110023

Kentisuchus spenceri

???????????????????????????????4?0??11011?0110?1210000???0??0100101001111?????00??200000???11??003

Dollosuchoides densmorei

001??111?00?0011?1?00?????1?0??4?000??01??012??1210000??00??01?0101001101??????1??2000001????000?3

Brachyuranochampsa eversolei

??????????????????????????????????????????011??1210000??00??0?0??01101010??0?000?1210000????100003

“Crocodylus” depressifrons

?????11010010011?110010??1110211?01000011?0110?1110000?110??0001001?01?10?102000012000001101100003

“Crocodylus” acer

??????????????????????????????????????????0110?1210000??00??000100?101010?00?0100?2000001??1100003

“Crocodylus” affinis

00111100000100110110010??11102111010000110011010110000?010??0001001101010??010?0012000001???100003

Asiatosuchus germanicus

001?1001?01?0?11?1??0????1110200?010??01?00010?001000???10??00010???01010??0100001110000????10?003

Prodiplocynodon langi

??????????????????????????????????????????0110??030000??10?00001001101?10??0?0?0?11?00001??0101003

Benton, M. J. and J. M. Clark. 1988 Archosaur phylogeny and the relationships of the Crocodylia; pp. 295-338 in M. J. Benton (ed.), The Phylogeny and Classification of the Tetrapods. Clarendon Press, Oxford.

Brochu, C. A. 1999. Phylogeny, systematics, and historical biogeography of Alligatoroidea. Society of Vertebrate Paleontology Memoir 6:9-100.

Brochu, C. A. 2004. Alligatorine phylogeny and the status of *Allognathosuchus* Mook, 1921. Journal of Vertebrate Paleontology 24:856-872.

Brochu, C. A. 2007. Morphology, relationships and biogeographic significance of an extinct horned crocodile (Crocodylia, Crocodylidae) from the Quaternary of Madagascar. Zoological Journal of the Linnean Society 150:835-863.

Buscalioni, A. D., J. L. Sanz, and M. L. Casanovas. 1992. A new species of the eusuchian crocodile *Diplocynodon* from the Eocene of Spain. Neues Jahrbuch für Geologie und Paläontologie Abandlungen 187:1-29.

Clark, J. M. 1994. Patterns of evolution in Mesozoic Crocodyliformes; pp. 84-97 in N. C. Fraser and H. D. Sues (eds.), In the Shadow of Dinosaurs: Early Mesozoic Tetrapods. Cambridge University Press, New York.

Norell, M. A. 1988. Cladistic approaches to paleobiology as applied to the phylogeny of alligatorids. Ph.D., Yale University, New Haven.

Norell, M. A. 1989. The higher level relationships of the extant Crocodylia. Journal of Herpetology 23:325-335.

Norell, M. A., and J. M. Clark. 1990. A reanalysis of *Bernissartia fagesii*, with comments on its phylogenetic position and its bearing on the origin and diagnosis of the Eusuchia. Bulletin de l'Institut Royal des Sciences Naturelles de Belgique 60:115-128.

Willis, P. M. A. 1993. *Trilophosuchus rakhami* gen et sp. nov., a new crocodilian from the Early Miocene limestones of Riversleigh, northwestern Queensland. Journal of Vertebrate Paleontology 13:90-98.
